# Observed to expected lung area to head circumference ratio (O/E LHR) in fetuses with congenital anomalies of the kidney and urinary tract (CAKUT): assessment and evaluation as predictive factor for acute postnatal outcome—a single center study

**DOI:** 10.3389/fped.2023.1145907

**Published:** 2023-06-19

**Authors:** M. Sourouni, L. Haisch, K. Oelmeier, M. Möllers, D. Willy, K. Sondern, H. Köster, J. Steinhard, J. Sandkötter, W. Klockenbusch, R. Schmitz, J. Potratz

**Affiliations:** ^1^ Department of Obstetrics and Gynecology, University Hospital Muenster, Muenster, Germany; ^2^ Department of Gynecological Endocrinology and Fertility Disorders, University Hospital Heidelberg, Heidelberg, Germany; ^3^ Department of Pediatrics, University Hospital Muenster, Muenster, Germany; ^4^ Gynaecologist’s Office of Dr. Natalia Ulrich, Berlin, Germany; ^5^ Department of Fetal Cardiology, University Hospital of the Ruhr University Bochum, Bad Oeynhausen, Germany

**Keywords:** pregnancy, ultrasound, prenatal medicine, CAKUT, LHR

## Abstract

**Introduction:**

To assess the observed to expected lung area to head circumference ratio (O/E LHR) in fetuses with congenital anomalies of the kidney and urinary tract (CAKUT) and to explore its value as a potential predictive factor for postnatal outcome.

**Methods:**

A retrospective single-center study was conducted on pregnancies complicated by CAKUT between 2007 and 2018. The lung-to-head ratio (LHR) was calculated for each fetus by two independent observers. Correlations between O/E LHR and various perinatal outcome factors were assessed with Spearman's rank correlation. Furthermore, nominal logistic regression was performed to assess O/E LHR as predictive factor for respiratory distress in newborn.

**Results:**

Of 64 pregnancies complicated by CAKUT, 23 were terminated. In the 41 cases of continuation of pregnancy, newborn presenting respiratory distress with need for respiratory support in the delivery room showed earlier gestational age at onset of amniotic fluid abnormalities and at birth. Although median O/E LHR and median single deepest pocket (SDP) of amniotic fluid were significantly smaller in newborn that did develop respiratory distress with need of respiratory support in the delivery room, neither O/E LHR nor SDP were accurate predictors for the development of respiratory distress.

**Conclusions:**

Our data show that O/E LHR alone cannot serve as a predictive marker for fetal outcome in pregnancies complicated by CAKUT, though it might still be a helpful parameter together with detailed renal ultrasound evaluation, onset of amniotic fluid abnormality and SDP, particularly in its extreme values.

## 1. Introduction

High-resolution ultrasound equipment provides the opportunity of identifying congenital anomalies prenatally. Detection and management of congenital malformations are essential aspects of perinatal medicine. The overall prevalence of congenital anomalies is approximately 1%–2% (1). The most common prenatally identified malformations are abnormalities of the genitourinary tract, with an incidence of 1–4 in 1,000 pregnancies (2). As such, they represent 15%–20% of all prenatally diagnosed congenital anomalies (3).

In congenital abnormalities of the kidney and genitourinary tract (CAKUT), the amount of amniotic fluid is generally variable and renal function can be impaired. The concomitant presence of oligohydramnios indicates significant global fetal renal dysfunction and is a risk factor for the development of pulmonary hypoplasia and poor outcome, depending on gestational age at occurrence (4, 5). Indeed, perinatal mortality in fetuses with LUTO is mainly attributed to end-stage renal disease and severe pulmonary hypoplasia as a result of severe oligohydramnios during the critical periods of lung development (6, 7). However, more recent studies report that oligohydramnios is not always associated with a poor prognosis and long-term outcome in survivors is encouraging (8).

Congenital diaphragmatic hernia (CDH) is a distinct clinical entity in which fetal pulmonary hypoplasia occurs, with severity of pulmonary hypoplasia providing an important marker in the prenatal prediction of postnatal survival. The most commonly used method for the indirect assessment of fetal lung volume in CDH is measurement of the lung-to-head-circumference ratio (LHR) (9, 10). The lung area can be obtained either by taking the product of the longest two perpendicular linear measurements of the lung measured at the level of the cardiac four-chamber view on a transverse scan of the fetal thorax or by manual tracing of the limits of the lung. The product is then divided by the head circumference to obtain the LHR. If the LHR is 1 or less, the prognosis is poor. As Peralta and associates showed that normal LHR increases exponentially with gestational age (11, 12), Jani and colleagues proposed the introduction of a new measurement, the observed to expected LHR (O/E LHR), to correct for gestational age (13). More recently, fetal magnetic resonance imaging (MRI) offers another, though less widely available tool to evaluate the severity of CDH. Fetal MRI facilitates evaluation by (a) ensuring accessibility independent of fetal position, (b) minimizing the effect of maternal and fetal motion, (c) allowing identification of associated anomalies and anatomical measurements with superior tissue contrast, (d) providing a large field of view and (e) relative operator independence (14). Moreover, it enables visualization and measurement of the ipsilateral as well as contralateral lung, so that the total lung volume (TLV) can be quantified. Interestingly, measurement of the contralateral lung area by 2D ultrasound correlates well with TLV determined by MRI, irrespective of gestational age, liver herniation or side of the defect (15). While fetal MRI could potentially have better predictive value regarding fetal outcome and a recent meta-analysis concluded that fetal MRI could be used to predict neonatal survival, data remain inconsistent and further research is required (16).

Although LHR and O/E LHR have been established as an indirect assessment of fetal lung volume in cases of CDH, no data has been published regarding the predictive value of LHR and O/E LHR in fetuses with CAKUT. In this group, solely abnormal amniotic fluid volume has been correlated to postnatal outcome with contradictory results (6–8).

The objective of the current study was a) to assess the O/E LHR in fetuses with CAKUT and b) evaluate its role as an indirect assessment of fetal lung volume in the prenatal prediction of postnatal outcome.

## 2. Methods

This retrospective cohort study was conducted at the Department of Obstetrics and Gynecology and the Department of Pediatrics of the University Hospital Muenster. The study was designed according to the Declaration of Helsinki and was approved by the institutional review board.

### 2.1. Inclusion criteria and data collection

Eligible patients were identified by searching the Department’s ultrasound database for cases of fetal CAKUT (*n* = 138) from 2007 to 2018. Data were collected from patient charts. We excluded cases with no data on delivery outcome, twin pregnancies and cases with prenatal diagnosis of genetic syndromes or complex congenital heart defects that would affect postnatal outcome (*n* = 45). Cases with no ultrasound data past the 20th week of pregnancy (*n* = 22) or cases with no data on newborn outcome (*n* = 7) were also excluded.

All ultrasound examinations were performed by advanced specialists in prenatal medicine certified by the German Association for Ultrasound in Medicine (DEGUM). The single deepest vertical pocket (SDP) of amniotic fluid was determined to estimate the amniotic fluid level. Oligohydramnios was defined as a SDP of 20 mm or less, anhydramnios as a SDP of 0 mm. Prenatal diagnosis of CAKUT was divided into three categories based on mechanism and expected severity of lung hypoplasia: a) bilateral renal agenesis (lung hypoplasia due to oligohydramnios), b) CAKUT with an obvious increase of intra-abdominal pressure (lung hypoplasia due to diaphragmatic displacement, e.g., cystic renal disease, hydronephrosis, megaureter or megacystis) or c) CAKUT without increase of intra-abdominal pressure (e.g., renal hypoplasia/dysplasia, kidney cysts). The allocation was made according to the prenatal diagnosis after review of the corresponding ultrasound images by two independent specialists. The right lung-to-head ratio (LHR) was calculated for each fetus by two independent specialists per ultrasound as described by Metkus et al. by multiplication of the longest diameter of the lung by its longest perpendicular diameter (9). The expected LHR was then calculated as described by Posada et al. (17) and the O/E LHR was calculated as Observed LHR/Expected LHR × 100. Primary endpoint of the study was development of respiratory distress with need for respiratory support in the delivery room. Secondary endpoints included umbilical cord arterial pH, Apgar score at 5 and 10 min and survival to hospital discharge.

### 2.2. Statistical analysis

Statistical analysis was performed using the Statistical Package for the Social Sciences (SPSS) software, version 28 (IBM Corporation, New York, NY, USA). Descriptive statistics were used to characterize the study population. Normally and non-normally distributed parameters were shown as median and interquartile range. All inferential statistics were intended to be exploratory (hypothesis generating), not confirmatory, and are interpreted accordingly. Therefore, no power calculation was performed. The *p*-values were considered significant if *p* ≤ 0.05. Correlations between O/E LHR and variables of interest, including gestational age at onset of amniotic fluid abnormality, SDP, gestational age at delivery, umbilical cord pH, 5 and 10 min Apgar score, development of respiratory distress with respiratory support in the delivery room, need for invasive ventilation and survival to hospital discharge were examined using Spearman's rank correlation coefficient *ρ* (rho). Nominal logistic regression analysis was used to assess the statistical significance of the continuous numerical variables O/E LHR, gestational age at delivery and SDP as prognostic factors for the development of respiratory distress with need for respiratory support in the delivery room.

## 3. Results

### 3.1. Description of study population

We identified 64 eligible patients. Of these, 41 women continued (group A) and 23 terminated their pregnancy (group B). Baseline characteristics and sonographic findings of both groups are shown in Table 1. For patient flow chart and detailed clinical data, please see Supplementary Figure S1 and Supplementary Table S1. In five pregnancies with prenatal suspicion of associated anomalies, postnatal diagnoses were VACTERL association (oesophageal atresia IIIB, anal atresia, VSD, choanal stenosis, clubfoot, *n* = 1), campomelic dysplasia (median cleft palate, retrognathia, dextrocardia, facial dysmorphism, sex reversal, *n* = 1), Wolf-Hirschhorn syndrome (*n* = 1), anal atresia (*n* = 1) and cleft lip and palate (*n* = 1). Seven additional newborn were postnatally diagnosed with associated malformations: prune belly syndrome (*n* = 2), Zuelzer-Wilson syndrome (*n* = 1), Caroli syndrome (*n* = 1), caudal regression syndrome (*n* = 1), anal atresia with congenital tapeto-retinal amaurosis (*n* = 1), Bardet-Biedl syndrome (*n* = 1).

**Table 1:** Characteristics of study population.

		Continuation of pregnancy, Group A (*n* = 41)	Termination of pregnancy, Group B (*n* = 23)	
Type of fetal CAKUT	Bilateral renal agenesis	14.6% (6/41)	56.5% (13/23)	
	CAKUT with increased intra-abdominal pressure	36.6% (15/41)	34.8% (8/23)	
	CAKUT with no increase in intra-abdominal pressure	48.7% (20/41)	8.7% (2/23)	
Suspected syndromic disease prenatally	No	87.8% (36/41)	82.6% (19/23)	
	Yes	12.2% (5/41)	17.4% (4/23)	
Amniotic fluid amount	Normal	34.1% (14/41)	4.3% (1/23)	
	Oligohydramnios Median SDP in mm (IQR)	22.0% (9/41) 17.00 (10.25–17.00)	34.8% (8/23) 18.00 (14.6–20.00)	
	Anhydramnios	39.0% (16/41)	60.9% (14/23)	
	Polyhydramnios	4.9% (2/41)	0.0% (0/23)	
Median gestational age in weeks at the onset of amniotic fluid disorder (IQR)		29.0 (20.0–31.0)	21.5 (20.0–22.0)	*p* = 0.004*
Median O/E LHR (IQR)		46.42% (34.73–57.44)	41.84% (31.81–54.53)	*p* = 0.561
Median gestational age in weeks at calculation of O/E LHR (IQR)		32.0 (28.0–35.5)	21.0 (20.0–23.0)	*p* < 0.001*

IQR, interquartile range.

*p < 0.05 was considered significant.

In group B, median gestational age at onset of amniotic fluid abnormality and at assessment of O/E LHR was lower than in group A. The mean O/E LHR did not differ significantly between clinical groups, but was lower in cases with bilateral renal agenesis (41.8%) and CAKUT with increased intra-abdominal pressure (42.9%) than in CAKUT without increased intra-abdominal pressure (45.7%, *p* = 0.13 and *p* = 0.25).

Our center did not support fetal interventions such as amnioinfusion, vesicoamniotic shunting (VAS) or bladder puncture. However, since our cohort comprised patients referred from other medical centers, there were eleven cases of fetal intervention. More specifically, group A included six cases of amnioinfusion, one case of VAS, one case of bladder puncture and one case of amnioinfusion combined with VAS. In group B, there were two cases of bladder puncture and one case of amnioinfusion combined with bladder puncture.

### 3.2. Outcome data of study population

The outcome of pregnancies of group A is described in Table 2 (for detailed clinical data also see Supplementary Table S1). Respiratory support in the delivery room was required in 23 cases (56.1%). Of these, 22 patients received ongoing support, with invasive ventilation in 17 cases (41.5%). High-frequency ventilation and inhaled nitric oxide were utilized in 16 (39.0%) and 12 (29.3%) patients, respectively, and seven (17.1%) patients received Surfactant.

**Table 2:** Outcome data of study population.

		Continuation of pregnancy, Group A (*n* = 41)	Termination of pregnancy, Group B (*n* = 23)
Live birth		95.1% (39/41)	17.4% (4/23)
Median gestational age at birth in weeks		37.0 (35.0–39.5)	23.0 (22.0–24.0)
Median birth weight in gramm, (IQR)/Voigt percentile (IQR)		2675 (2052–3637)/56 (9–79)	477 (385–665)/45 (16–69)
Birth modus	Vaginal	36.6% (15/41)	17.4% (4/23)
	Sectio caesarea	58.5% (24/41)	0.0% (0/23)
	Stillbirth	4.9% (2/41)	82.6% (19/23)
Respiratory distress with need for respiratory support in the delivery room	Yes	56.1% (23/41)	n/a
	No	43.9% (18/41)	n/a
Ongoing respiratory support beyond delivery room	Yes	53.6% (22/41)	n/a
	No	46.4% (19/41)	n/a
Pneumothorax		12.2% (5/41)	n/a
Relevant associated congenital anomalies		29.3% (12/41)	n/a

n/a, not applicable.

IQR, interquartile range.

None of the outcome parameters showed a strong (rho ≥ 0.6, *p* ≤ 0.05) correlation to O/E LHR (Table 3). However, O/E LHR showed a weak negative (rho = −0.343, *p* = 0.033) correlation with the development of respiratory distress with need for respiratory support in the delivery room. A strong (rho = −0.7) correlation of lower O/E LHR with need for invasive ventilation did not reach significance (*p* = 0.782). In contrast, the SDP showed a moderate (rho = 0.454) yet significant (*p* = 0.034) correlation with higher O/E LHR. Moderate to strong meaningful correlation (rho ≥ 0.6, *p* ≤ 0.05) was found between gestational age at onset of amniotic fluid abnormality and 5 and 10 min Apgar scores as well as between the gestational age at birth and 5 and 10 min Apgar scores.

**Table 3:** Spearman correlation coefficient analysis of O/E LHR in ongoing pregnancies complicated by CAKUT.

Variable	Correlation coefficient (*ρ*)*	*P*-value**
Gestational age at onset of amniotic fluid abnormality (*n* = 27)	0.156	0.437
SDP (*n* = 9)	0.454	0.034**
Gestational age in weeks at birth (*n* = 41)	0.193	0.228
Umbilical cord arterial pH (*n* = 38)	−0.123	0.463
5’ Apgar (*n* = 39)	0.207	0.213
10’ Apgar (*n* = 39)	0.227	0.170
Respiratory distress syndrome with need for respiratory support in delivery room (*n* = 41)	−0.343	0.033**
Patient with need for invasive ventilation beyond delivery room (*n* = 17)	−0.70	0.782
Survival to discharge from hospital (*n* = 24)	0.274	0.096

Value spectrum: ±0.6 – 0.8 (strong association), ±0.4 – 0.6 (moderate association), ±0.2 – 0.4 (weak association), ±0 – 0.2 (very weak association).

* Positive or negative ρ values imply a positive or negative association, respectively.

** *p* ≤ 0.05 was considered significant.

**Figure 1:**
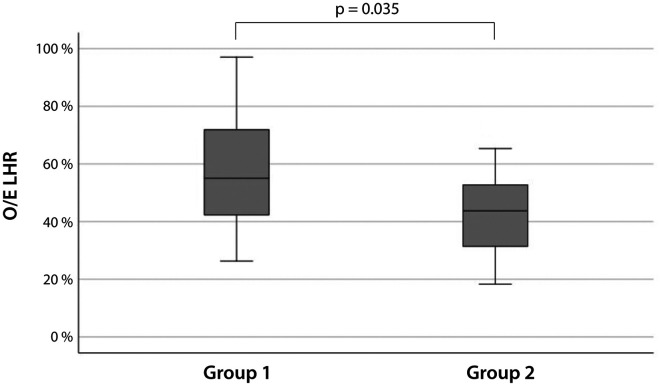
O/E LHR of newborns with CAKUT that did not (left, group 1) or did (right, group 2) develop respiratory distress with immediate need for respiratory support.

Median O/E LHR, median SDP, median gestational age at the onset of amniotic fluid abnormality and at birth differed significantly between newborn that did not require respiratory support during delivery room management and those that did (Table 4). The median O/E LHR was higher [55.0% (41.7–75.0) vs. 43.7% (31.0–53.0); *p* = 0.035] in the first group (Figure 1). However, regression analysis demonstrated that gestational age at birth was a significant predictor of respiratory distress (*p* = 0.004), whereas O/E LHR was not (*p* = 0.105). The SDP could also not reliably predict need for respiratory support (*p* = 0.073).

**Table 4:** Characteristics of newborn with CAKUT that did not (left, group 1) or did (right, group 2) develop respiratory distress with immediate need for respiratory support.

	Group 1 (*n* = 16)	Group 2 (*n* = 23)
Median O/E LHR (IQR)	55.0% (41.74–75.0)	43.7% (31.0–53.0), *p* = 0.035*
Median SDP (IQR)	27.0 mm (9–99.5)	0.0 mm (0.0–8.0), *p* = 0.002*
Median gestational age in weeks at the onset of amniotic fluid disorder (IQR)	34.0 (31.0–38.5)	26.0 (20.0–29.0), *p* < 0.001*
Median gestational age in weeks at birth (IQR)	40.0 (39.0–41.7)	36.0 (34.0–38.0), *p* < 0.001*

*p < 0.05 was considered significant.

Median O/E LHR did not differ significantly between newborn with no need for respiratory support in the delivery room and newborn with ongoing need for invasive ventilation [55.0% (41.7–75.0), 41.9% (29.9–53.3), *p* = 0.464]. Although O/E LHR was higher in survivors [51.6% (35.3–63.2)] compared with non-survivors [41.6% (31.0–50.0)], the difference was not statistically significant (*p* = 0.218).

### 3.3. Follow up

Of 39 live births, 15 newborn (38.5%) died before discharge from hospital (Supplementary Figure S1, Supplementary Table S1). Renal diagnoses were bilateral renal agenesis (*n* = 4), CAKUT with increased intra-abdominal pressure (*n* = 8) and CAKUT without increase in intra-abdominal pressure (*n* = 3). Median survival was 0.11 (0–0.13) days, 2.55 (0.73–4.74) days and 0.13 (0.11–0.47) days respectively and not significantly different between the groups (*p* > 0.05). Two newborn received primary palliative care. Of 13 newborn with intensive care management, one patient stabilized on non-invasive ventilation, but died on day 7 due to complications of arterial hypertension. The other 12 newborn required invasive mechanical ventilation (median minimum mean airway pressure of 14 cmH_2_O) and died on secondary palliative care due to persistent pulmonary (*n* = 9) or renal (*n* = 3) failure. Three patients received renal replacement therapy (RRT; peritoneal dialysis *n* = 1, continuous veno-venous hemofiltration *n* = 2).

Twenty-four (61.5%) patients survived to hospital discharge (Supplementary Figure S1, Supplementary Table S1). Renal diagnoses in these patients were bilateral renal agenesis (*n* = 1), CAKUT with increase in intra-abdominal pressure (*n* = 7) and CAKUT without increase in intra-abdominal pressure (*n* = 16). All 16 patients that did not require respiratory support in the delivery room were amongst survivors, as well as five newborn that had required invasive ventilation. Three newborn required RRT (peritoneal dialysis *n* = 3). One patient was discharged on secondary palliative care (bilateral renal agenesis and Zuelzer-Wilson syndrome), two additional patients died at 4.5 and 14.6 month due to sepsis (both CAKUT without increase of intra-abdominal pressure and additional malformations). Twenty-one (53.8%) patients are alive with a median follow-up of 74.2 (16.8–107.4) months. At 3.0 and 4.7 years of age, two additional patients required RRT (peritoneal dialysis). Three patients have received a kidney transplant.

## 4. Discussion

Diagnosis of congenital malformations, detailed counseling and support of the parents and prenatal therapy (if possible) are essential tasks of perinatal medicine. Thus, the prenatal management of CAKUT aims to describe the type of anomaly as accurately as possible, detect associated malformations and screen for parameters predictive of renal function, as well as counsel parents on the child’s perspective clinical course and prognosis in a multidisciplinary approach (18). Antenatal ultrasound parameters predictive of renal function and respiratory distress can support counseling and decision-making in regard to pregnancy termination, prenatal procedures and delivery room management after birth (19). As poor perinatal outcome has been attributed to pulmonary hypoplasia, oligohydramnios has previously been examined as a predictive factor, with contradictory results (8). Nevertheless, the predictive potential of other factors related to lung hypoplasia such as the O/E LHR, which is well established for prediction of lung hypoplasia in CDH (9, 10), has not been previously evaluated in CAKUT.

Because pregnancies complicated by CAKUT can have a poor outcome, with fetal and neonatal survival at risk and imminent intensive medical needs, parents experience immense emotional strain and termination of pregnancy is frequently practiced in this situation (6). This is in striking contrast to the dramatic progress that has been made in neonatal intensive care including pulmonary management. In our cohort, 23 couples decided to terminate the pregnancy after counseling. In this group the median O/E LHR was lower, yet not significantly so. An earlier gestational age at assessment of O/E LHR as well as at detection of amniotic fluid abnormality indicate the severity of underlying disease in these cases.

In continued pregnancies, younger gestational age at birth and at onset of amniotic fluid abnormality seemed to predict the development of respiratory distress as well as lower 5’ and 10’ minute Apgar scores in newborn with CAKUT. This is in keeping with multiple reports that associate preterm birth (20–22) and/or oligohydramnios (23–25) with poor perinatal outcome. However, O/E LHR alone did not prove to be a credible prognostic marker for predicting poor outcome in our cohort. Still, a median O/E LHR of 43.9% (33.5–57.1) overall underlines that in comparison to healthy fetuses, fetuses with CAKUT present with impaired lung development and pulmonary hypoplasia. Furthermore, O/E LHR values corresponded, though not statistically significant, to clinical categories of severity, with lower values in bilateral renal agenesis and CAKUT with increased intra-abdominal pressure compared to those cases without (41.8% and 42.9% respectively, compared to 45.7%). Similarly, O/E LHR appeared lower in neonates requiring invasive ventilation compared to non-invasive respiratory support (41.9% vs. 49.5%) and in non-survivors compared to surviving infants (41.6% vs. 51.6%). These data support O/E LHR as an additional but not alone-standing predictive marker of perinatal outcome. For comparison, in CDH, where O/E LHR serves as an established prognostic marker, O/E LHR discriminates non-survivors and survivors with mean values of 30.3% and 44.2% (*p* < 0.01), respectively, and a 100% survival reported at O/E LHR of >45% (26, 27). Such different mean O/E LHR values may reflect the distinct pathophysiology and a different relationship between lung volume on imaging and functional deficit in these two conditions complicated by pulmonary hypoplasia. On the other hand, it must be noted that death of CAKUT patients in our cohort was not uniformly due to lung hypoplasia (*n* = 9; mean O/E LHR 35.7%), but also due to other causes (*n* = 6).

The main limitation of our work is a limited sample size of pregnancies continued after diagnosis of CAKUT (*n* = 41). Clinically distinct subgroups might not be sufficiently represented and the possible predictive value of O/E LHR in fetuses with CAKUT may be underrated. For instance, cases with expected severe pulmonary hypoplasia due to ahydramnios were more frequent in the group that terminated pregnancy (60.9% vs. 39.0%) and the median gestational age of onset of oligohydramnios in this group was significantly earlier (21.5 vs. 29.0 weeks, *p* = 0.004). Such ’selection bias’ may mask the value of O/E LHR as prognostic marker of perinatal outcome.

In our cohort, fetal intervention in form of amnioinfusion, vesicoamniotic shunting (VAS), bladder puncture or combination thereof was performed in eleven cases. The procedure of amnioinfusion is considered to be safe and one of the few available methods in the active management of pregnancies affected by oligohydramnios (28–30). Available data support an advantage regarding perinatal morbidity and mortality (27). Moreover, it has been shown to improve diagnostic accuracy by enabling a more comprehensive ultrasonographic evaluation in severe oligohydramnios (31). VAS also appears to be advantageous for perinatal compared to conservative management (7, 32). However, longer-term survival and outcome of renal function after VAS procedure remain uncertain (7) and further study is required.

The strengths of this work include its high quality ultrasound data, collected by advanced specialists in prenatal medicine, and its novel evaluation of O/E LHR, an established and validated parameter in other prenatal settings (11, 12), for CAKUT, a group of diagnoses contributing considerably to peri- and postnatal morbidity and mortality. In addition, we provide granular data on pulmonary and renal outcome of affected neonates that may aid prenatal parental counselling.

In conclusion, O/E LHR alone does not serve as a sufficient predictor of perinatal outcome in pregnancies complicated by CAKUT. Prenatal counseling should therefore continue to focus on renal ultrasound, gestational age at onset and severity of amniotic fluid abnormality and the risk of preterm delivery as best-established predictive markers. However, further prospective studies are needed to substantiate and expand our current knowledge. Here, O/E LHR may provide an interesting candidate marker, particularly in its extreme values.

## Data Availability

The raw data supporting the conclusions of this article will be made available by the authors, without undue reservation.
