# Supplementary figures and images for: Observed to expected lung area to head circumference ratio (O/E LHR) in fetuses with congenital anomalies of the kidney and urinary tract (CAKUT): assessment and evaluation as predictive factor for acute postnatal outcome—a single center study
Source: Front Pediatr. 2023 Jun 19;11:1145907. doi: 10.3389/fped.2023.1145907 (PMC10317208; doi:10.3389/fped.2023.1145907)

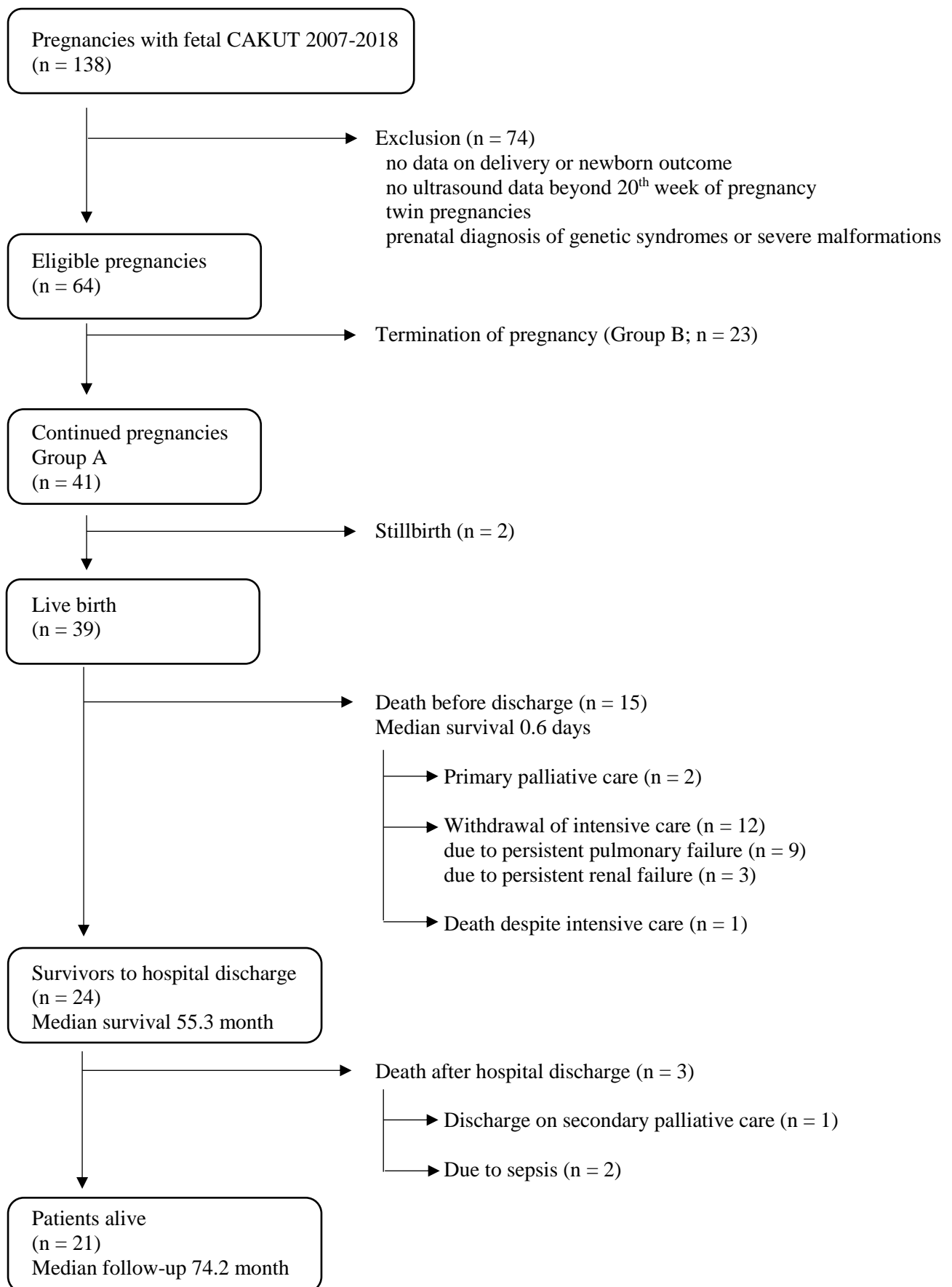

**Supplementary Figure 1.** Patient flowchart

Supplement: Supplementary file 1 [file Image1.pdf]
